# MicRhoDE: a curated database for the analysis of microbial rhodopsin diversity and evolution

**DOI:** 10.1093/database/bav080

**Published:** 2015-08-18

**Authors:** Dominique Boeuf, Stéphane Audic, Loraine Brillet-Guéguen, Christophe Caron, Christian Jeanthon

**Affiliations:** ^1^CNRS, UMR 7144, Marine Phototrophic Prokaryotes Team,; ^2^Sorbonne Universités, UPMC Univ Paris 06, UMR 7144, Oceanic Plankton Group,; ^3^CNRS, UMR 7144, Team Evolution des Protistes et Ecosystèmes Pélagiques and; ^4^CNRS, UPMC, FR2424, ABiMS, Station Biologique de Roscoff, F-29680 Roscoff, France

## Abstract

Microbial rhodopsins are a diverse group of photoactive transmembrane proteins found in all three domains of life and in viruses. Today, microbial rhodopsin research is a flourishing research field in which new understandings of rhodopsin diversity, function and evolution are contributing to broader microbiological and molecular knowledge. Here, we describe MicRhoDE, a comprehensive, high-quality and freely accessible database that facilitates analysis of the diversity and evolution of microbial rhodopsins. Rhodopsin sequences isolated from a vast array of marine and terrestrial environments were manually collected and curated. To each rhodopsin sequence are associated related metadata, including predicted spectral tuning of the protein, putative activity and function, taxonomy for sequences that can be linked to a 16S rRNA gene, sampling date and location, and supporting literature. The database currently covers 7857 aligned sequences from more than 450 environmental samples or organisms. Based on a robust phylogenetic analysis, we introduce an operational classification system with multiple phylogenetic levels ranging from superclusters to species-level operational taxonomic units. An integrated pipeline for online sequence alignment and phylogenetic tree construction is also provided. With a user-friendly interface and integrated online bioinformatics tools, this unique resource should be highly valuable for upcoming studies of the biogeography, diversity, distribution and evolution of microbial rhodopsins.

**Database URL**: http://micrhode.sb-roscoff.fr.

## Introduction

Rhodopsins are photochemically active membrane proteins that are composed of seven transmembrane helices with a retinal chromophore. According to their amino acid sequences, they are divided in two families known as either type-1 rhodopsins, that are all of microbial origin or type-2 rhodopsins that are animal photosensitive receptors (1). Type-1 rhodopsins include light-driven proton pumps (e.g. bacteriorhodopsins and proteorhodopsins), ion pumps and channels, and light sensors. The first identified microbial rhodopsin, bacteriorhodopsin, was discovered from the cell membrane of the halophilic archaeon *Halobacterium salinarum* more than 40 years ago (2). Rhodopsins functioning as light-driven chloride pumps (halorhodopsins) with positive and negative phototactic sensors (sensory rhodopsins I, II and III) were further found in the same organism (3–5).

In 2000, a survey of total community DNA from Monterey Bay surface waters led to the discovery of a novel type of bacterial rhodopsin found in an uncultured marine gammaproteobacterium (6). Proteorhodopsin-mediated phototrophy is now known in a large variety of Bacteria and Archaea from diverse environments and lateral gene transfer most likely played an important role in their wide distribution across marine prokaryotes (7). Proteorhodopsin-containing microorganisms are widespread in terrestrial (soils, crusts, phyllosphere), freshwater (lakes, rivers, ponds, ice) and marine (including sea ice, hypersaline and brackish) photic environments (8–11). Recently, proteorhodopsin homologs were also detected in giant viruses that infect unicellular aquatic eukaryotes (12, 13). Proteorhodopsin acts as a proton pump (6, 14, 15) and could be involved as a secondary source of energy in the metabolism of heterotrophic prokaryotes through ATP generation (16, 17). Based on the analysis of marine and terrestrial metagenomic data, Finkel *et al.* (18) suggested that microbial rhodopsins are the prominent phototrophic mechanism on Earth. However, more investigations are needed to understand the physiological functions and fitness benefits of these proteins and their actual role in microbial ecology and in energetic balance of ecosystems.

In recent years, environmental genomics surveys have increasingly demonstrated the remarkable diversity of microbial rhodopsins in diverse aquatic and terrestrial environments (8–10, 12, 13, 19–31). Most of these studies have been performed by using the proteorhodopsin gene as molecular marker. Analyses of microbial gene sequences that serve as markers are facilitated by the availability of annotated databases of aligned sequences. Aligned sequences are required for diversity and phylogenetic analyses and for the design and evaluation of polymerase chain reaction (PCR) primers and probes. Group-specific PCR primers are used in quantitative real-time PCR for the quantification of gene copy numbers in the environment and for expression studies.

Here, we present MicRhoDE, a comprehensive, high-quality and freely accessible resource of nucleic acid sequences coding for microbial rhodopsins. The database and its associated description will be useful for studying the diversity, phylogeny and evolution of rhodopsin-containing microorganisms.

### Data collection and curation

**Figure 1.**
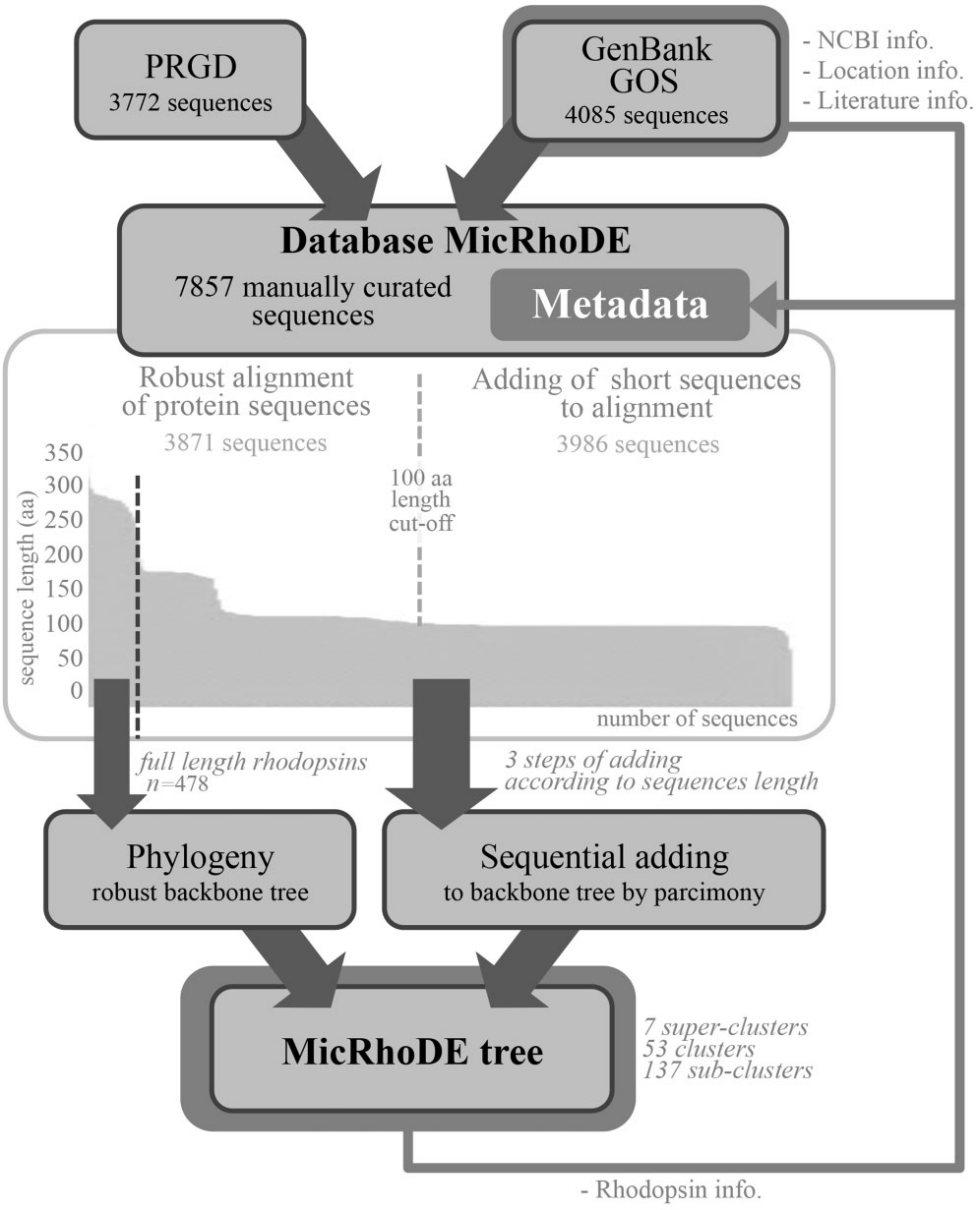
Flowchart of data in the MicRhoDE database. Arrows indicate sequence and metadata flows.

The MicRhoDE database was initially constructed by extracting reference proteorhodopsin sequences from GenBank (32), Global Ocean Sampling (GOS) database obtained from the CAMERA website (http://camera.crbs.ucsd.edu/) and from the literature (Figure 1). This initial set was further complemented with other type-1 rhodopsins (actinorhodopsins, xanthorhodopsins, bacteriorhodopsins, halorhodopsins and sensory rhodopsins) and newly discovered types (33, 34). To this initial set of sequences was added an original dataset (ProteoRhodopsin Global Diversity, PRGD) of marine proteorhodopsin genes obtained by Illumina sequencing of amplicons from diverse marine regions. The whole dataset was then used as a diversified seed to perform exhaustive similarity searches using BLAST in GenBank (as of March 2013) or GOS databases. BLAST results were dereplicated and manually checked for quality. Finally, all reference nucleic acid sequences were manually curated and modified from their original deposits in GenBank and GOS databases, when necessary, to be all in the same open reading frame. Because all MicRhoDE sequences are also stored in GenBank and GOS databases, NCBI or JCVI record IDs are also available in MicRhoDE to keep track of the original data source.

To date, 7857 type-1 rhodopsin sequences are stored in MicRhoDE, most of which (7193 sequences) represent proteorhodopsins. Although the majority of sequences are derived from environmental surveys of proteorhodopsin genes, the database also contains sequences obtained from a range of isolates or large genomic DNA fragments bearing a 16S rRNA gene copy. Among the 295 sequences whose taxonomic affiliation can be inferred from a 16S rRNA gene, 186 sequences come from cultivated organisms. Most sequences come from marine (93%) or freshwater environments (6%).

### Alignment and phylogenetic affiliation

**Figure 2.**
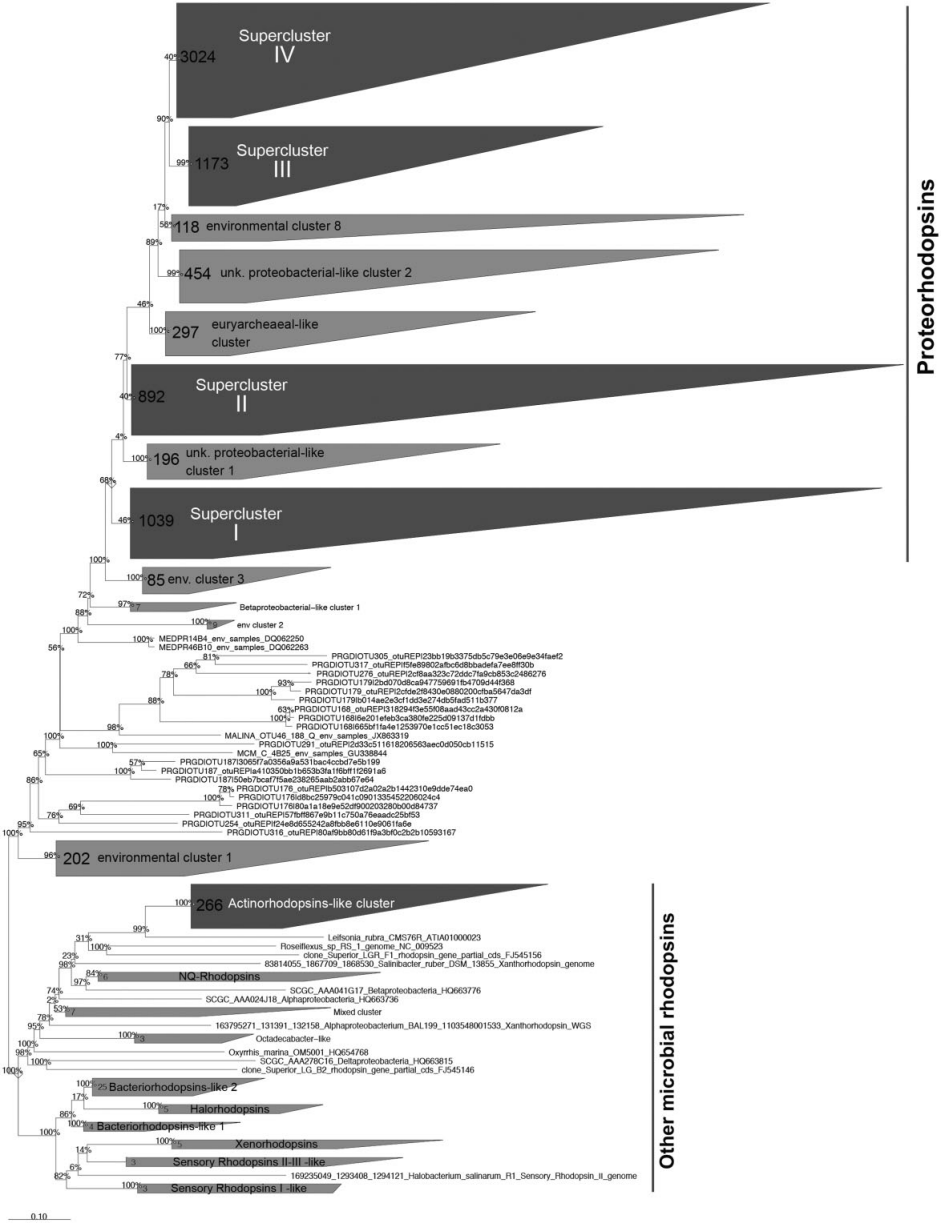
Phylogenetic relationships between the microbial rhodopsins stored in the MicRhoDe database. Numbers at the nodes are bootstrap values obtained by maximum parsimony. Numbers in clusters indicate the number of affiliated sequences.

Although lateral gene transfers and duplication events are prominent processes for the diversification of microbial genes among the three domains of life, we constructed a reference phylogenetic tree of microbial rhodopsins to allow a presumptive classification. Despite type-1 and type-2 rhodopsins share structural and functional similarities, there is a very low sequence identity between these two families (1). The seven transmembrane α-helices form a pocket in which the retinal, a vitamin-A aldehyde chromophore, is bound to a lysine residue by a *Schiff*-base linkage (35). This structure implies that evolutionary constraints vary according to the protein region. As a consequence, putative structure of the protein has to be considered when nucleic acid sequences are aligned. Since aligning 7857 nucleic acid sequences according to the secondary structure of the corresponding proteins is time-consuming, the sequence dataset was split in two parts (Figure 1). The 3871 longest amino acid sequences (>100 amino acid residues) were aligned according to the protein secondary structure using MAFFT eINSi strategy (36). The shorter ones were added to the robust alignment using MAFFT FFT strategy with the ‘–addfragments’ option that conserves the original alignment. The 478 full-length type-1 rhodopsin sequences of the database, including 86 strains and 73 different species, were used to construct a robust backbone tree (Figure 1) by Bayesian inference (4 Markov Chain Monte Carlo chains of 150 million generations) using PhyloBayes software (37). Shorter sequences were then sequentially inserted into the backbone tree by using the parsimony add option of the ARB software (38). The resulting tree (Figure 2) allowed us to establish a comprehensive classification system consisting in 5 superclusters, 53 clusters and 137 subclusters.

### Available metadata

The associated metadata of each sequence such as the sampling date, location, biome of origin, oceanic province were extracted from the related literature, GenBank and GOS records, checked manually, and reconciled before importation in the database. For each sequence are also provided the position in the phylogenetic tree, its NCBI taxonomy when available, the type of rhodopsin, its predicted spectral tuning according to the amino acid residue at position 105 for proteorhodopsins (15, 39) (Supplementary Figure S1). Putative activity and function according to the residues at position 97 and 108, respectively (40) and residue 101 for flavobacterial NQ rhodopsins (34) are also indicated. Altogether, the diversity of metadata associated to aligned and unaligned nucleic acid and protein sequences allows a variety of search options and data outputs.

### MicRhoDE web interface

**Figure 3.**
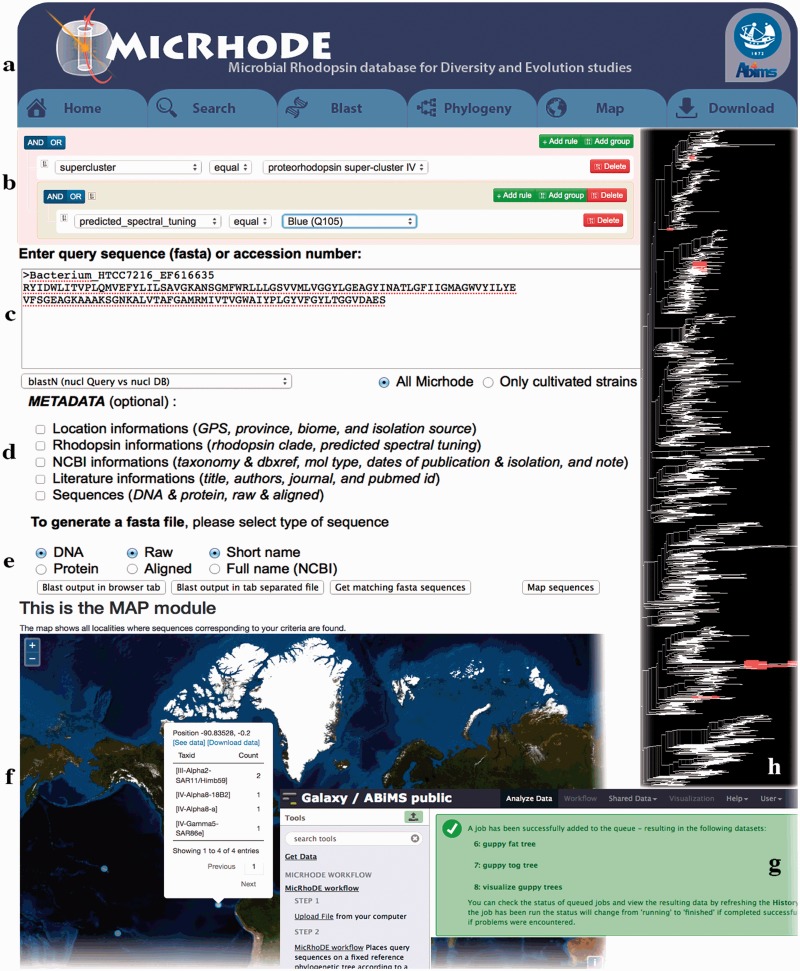
Screenshots of the MicRhoDE web interface showing the main content panel (**a**), the search (**b**) and (**c**) forms, the metadata (**d**) and output (**e**) options, a view of the map output option (**f**), the Galaxy instance for phylogenetic analysis (**g**) and an example of phylogenetic tree output (**h**).

MicRhoDE is a freely accessible public database (http://micrhode.sb-roscoff.fr) implemented using the perl Catalyst web framework (http://www.catalystframework.org/) backed by a PostgreSQL database (http://www.postgresql.org/). MicRhoDE will be updated annually by adding new type-1 rhodopsin gene sequences. In addition to a short introduction to microbial rhodopsins, the homepage provides a brief description of the database content and clickable icons with direct links to the major utilities of the database, including database and sequence similarity searching, and phylogeny (Figure 3a). MicRhoDE provides a powerful search module that accepts complex searches of given taxonomy, predicted protein features (such as activity, function and spectral tuning) and of a range of features (Figure 3b). Using a menu list, filters are also accessible for combining searches at the different cluster levels and features such as e.g. taxonomy, marine province of origin and predicted spectral tuning. Sequence similarity searches within the database are available using BLAST (version 2.2.26+) submission form in the BLAST page (Figure 3c). Metadata available in other public databases (accession ID, NCBI taxonomy, location, biome, marine province, date of isolation and related literature) and other restricted to MicRhoDE (rhodopsin affiliation according to phylogeny, rhodopsin type, predicted spectral tuning, putative activity and function) are optionally accessible in the data outputs of both search and BLAST modules (Figure 3d). Available data outputs include visualization of results on a map (Figure 3e and f).

The phylogeny page provides three different items: (i) a software pipeline proposing the user to place its own sequences into the type-1 rhodopsin reference phylogenetic tree, (ii) a schematic representation of the reference phylogenetic tree, highlighting the classification in clusters and superclusters and (iii) a detailed reference phylogenetic tree, displayed using the Archaeopteryx phylogenetic tree viewer Java applet (41). To place query amino acid sequences in the reference phylogeny, the user is redirected to a dedicated Galaxy instance (42–44) where the MicRhoDE workflow performs phylogenetic placement using Bayesian inference as implemented in the pplacer software (45). The Galaxy instance (Figure 3g) is available at http://webtools.sb-roscoff.fr/root?tool_id=abims_micrhode_workflow. Output files are visualized using the guppy program (a companion program of pplacer). Guppy (http://matsen.github.io/pplacer/generated_rst/guppy.html#) generates the phylogenetic tree showing either the probability of placements (fat visualization) or the best placements (tog visualization) of query sequences. The Galaxy framework provides interoperability mechanisms to dynamically call external viewer. Trees are generated in the phyloXML format and displayed using the Archaeopteryx phylogenetic tree viewer java applet (41) (Figure 3h).

To provide an intuitive overview of the geographic distribution of current data, the Map page displays for each location, the number of sequences available in MicRhoDE, the actual number of superclusters and clusters according to phylogeny and the dominant ones as well as the proportion of predicted spectral variants. The download page allows the download of the raw and aligned sequences of the database, their associated metadata, phylogenetic trees as well as a complete version of MicRhoDE formatted for the ARB software.

## Conclusion

MicRhoDE is a specialized database devoted to the study of microbial rhodopsins, which are functionally versatile proteins of crucial importance in the ecology of terrestrial and aquatic photic environments. As microbiologists from all fields use molecular, genomic and metagenomic methods to look at microbial diversity in the biosphere in more breadth and depth, we anticipate that the release of MicRhoDE will help comprehensive ecological and evolutionary analyses of these cosmopolitan genes.

## Supplementary Data

Supplementary data are available at *Database* Online.

Supplementary Data
