# Supplementary material for: MicRhoDE: a curated database for the analysis of microbial rhodopsin diversity and evolution
Source: Database (Oxford). 2015 Aug 18;2015:bav080. doi: 10.1093/database/bav080 (PMC4539915; doi:10.1093/database/bav080)
Supplement: Supplementary Data [file supp_2015_bav080_index.html]

MicRhoDE: a curated database for the analysis of microbial rhodopsin diversity and evolution — Supplementary Data 

# MicRhoDE: a curated database for the analysis of microbial rhodopsin diversity and evolution

## Supplementary Data

files

- Supplementary Data - zip file
