# Supplementary material for: MicRhoDE: a curated database for the analysis of microbial rhodopsin diversity and evolution
Source: Database (Oxford). 2015 Aug 18;2015:bav080. doi: 10.1093/database/bav080 (PMC4539915; doi:10.1093/database/bav080)
Supplement: Supplementary Data [file supp_bav080_Supp.zip › New Microsoft Office Word Document.docx]

**Supplementary Figure 1.** Distribution of microbial rhodopsins according to MicRhoDE exclusive features: classification in superclusters and clusters, predicted spectral tuning (dark blue: blue tuning, green: green tuning, grey: unknown or non-applicable). According to Fuhrman *et al*. (16), proteorhodopsins were classified as blue (Q variants)- and green (L, M, V, and A variants)-absorbing proteorhodopsins.
