# Supplementary figures and images for: MicRhoDE: a curated database for the analysis of microbial rhodopsin diversity and evolution
Source: Database (Oxford). 2015 Aug 18;2015:bav080. doi: 10.1093/database/bav080 (PMC4539915; doi:10.1093/database/bav080)

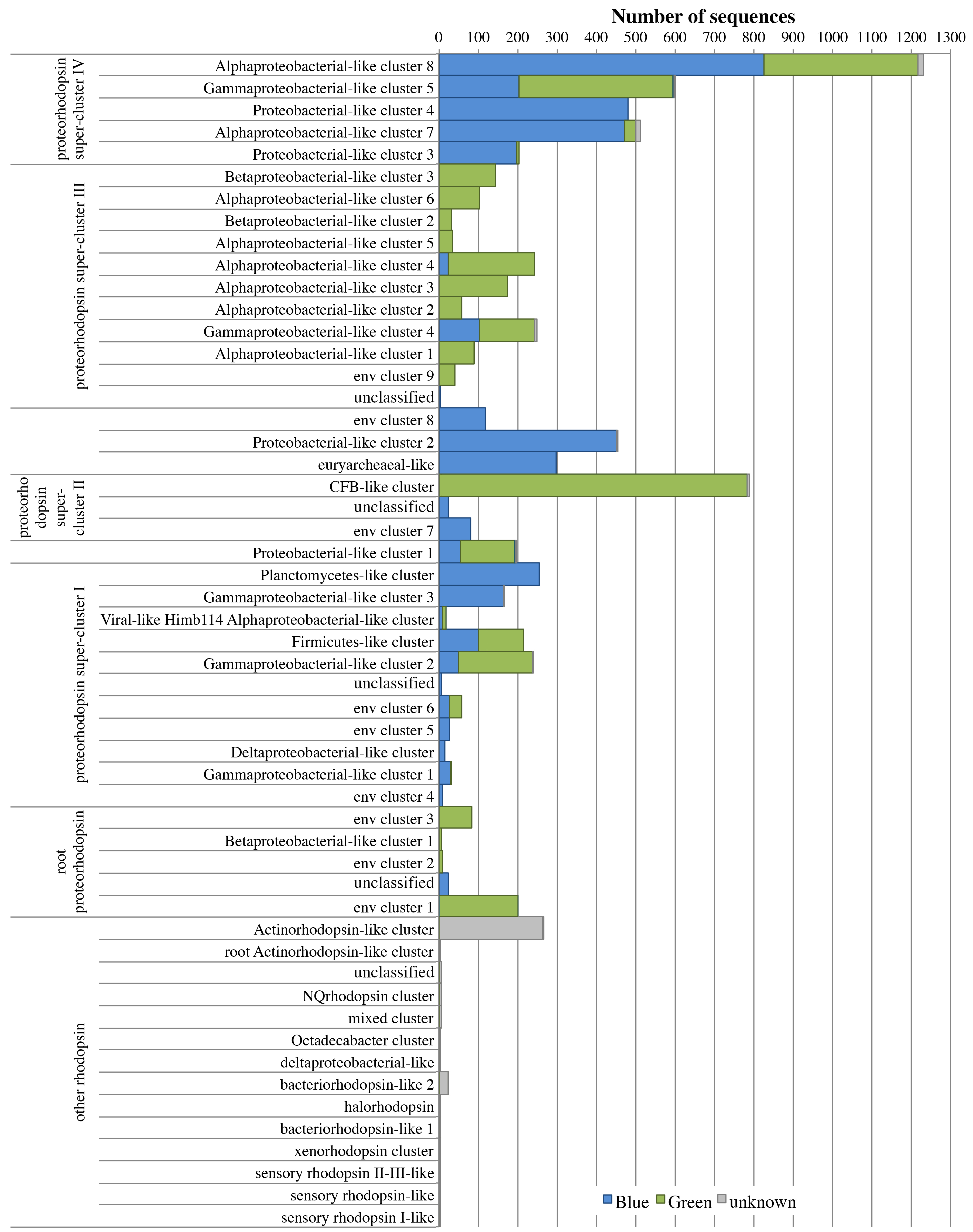

Supplement: Supplementary Data [file supp_bav080_Supp.zip › Supp. Figure 1.jpg]
